# Singularity-Free Neural Control for the Exponential Trajectory Tracking in Multiple-Input Uncertain Systems with Unknown Deadzone Nonlinearities

**DOI:** 10.1155/2014/951983

**Published:** 2014-06-19

**Authors:** J. Humberto Pérez-Cruz, José de Jesús Rubio, Rodrigo Encinas, Ricardo Balcazar

**Affiliations:** ^1^Sección de Estudios de Posgrado e Investigación, ESIME UA-IPN, Avenida de las Granjas, No. 682, Colonia Santa Catarina, México, DF 02250, Mexico; ^2^Departamento de Control Automático, CINVESTAV-IPN, Avenida Instituto Politécnico Nacional, No. 2508, México, DF 07360, Mexico

## Abstract

The trajectory tracking for a class of uncertain nonlinear systems in which the number of possible states is equal to the number of inputs and each input is preceded by an unknown symmetric deadzone is considered. The unknown dynamics is identified by means of a continuous time recurrent neural network in which the control singularity is conveniently avoided by guaranteeing the invertibility of the coupling matrix. Given this neural network-based mathematical model of the uncertain system, a singularity-free feedback linearization control law is developed in order to compel the system state to follow a reference trajectory. By means of Lyapunov-like analysis, the exponential convergence of the tracking error to a bounded zone can be proven. Likewise, the boundedness of all closed-loop signals can be guaranteed.

## 1. Introduction

During the last two decades, the control of systems using artificial neural networks (ANNs) has emerged as an effective and successful alternative to the conventional control techniques. The success of this approach lies on the universal approximation capability of ANNs which avoids the need for very time-consuming first principles modeling. Thus, it is possible to handle a broad class of nonlinear uncertain systems with little or (ideally) no a priori information.

The first deep insight about the identification and control of dynamic systems based on neural networks was provided by Narendra and Parthasarathy in [1]. However, they could not present a systematic procedure to analyze the stability of their neurocontrollers. This issue was addressed by Polycarpou and Ioannou [2], Rovithakis and Christodoulou [3], Kosmatopoulos et al. [4], and Yu and Poznyak [5]. They used Lyapunov-like analysis systematically in order to prove the stability of their algorithms. Based on these results, further refinements and improvements were accomplished in [6–9] and different applications were explored for robotics [10, 11], manufacturing systems [12], chemical process [13], power systems [14], and so on. It is worth mentioning that the vast majority of these studies are based on feedback linearization techniques. An inherent problem associated with these techniques is the possibility of the control singularity. A first approach to try to solve this problem is simply to focus only on a class of systems in which the gain function is known and constant [15, 16]. Certainly, to consider only this kind of systems could result into being very restrictive in practice. A generalized procedure to handle the control singularity consists of making modifications to the conventional adaptive algorithms [17–21]. Nonetheless, such modifications could provoke discontinuities in the control signal or well they could require the use of projection techniques. In this last case, the design and implementation process of such controllers could become quite complicated. To avoid the utilization of the projection, an integral-type Lyapunov function was proposed in [22]. On the basis of this function, a singularity-free smooth adaptive neural controller was developed. Notwithstanding, due to the requirement of the integral operation, the practical implementation of this approach is difficult [23]. In [24, 25], the neurocontrol of systems with a unique input was studied. The singularity was avoided by maintaining the input weight always different from zero. However, a systematic procedure for this goal was not specified.

Note that for the case of systems with multiple inputs, in particular when the number of states is equal to the number of inputs, the avoidance of the singularity cannot be guaranteed only by maintaining the coupling matrix of the neural network (see (12)), that is, *S*(*t*)*ϕ*(*x*(*t*)), different from zero. Evidently, a stronger condition is required. In fact, the necessary and sufficient condition to guarantee the nonsingularity is that the coupling matrix should always be invertible. To simplify the implementation of this condition, the input weight matrix *S*(*t*) and the sigmoidal function matrix *ϕ*(*x*(*t*)) can be constructed as square matrices. Besides, *ϕ*(*x*(*t*)) can be selected in such a way that its invertibility can be assured. Then, the problem now is focused on guaranteeing the invertibility of the input weight *S*(*t*). In this paper, unlike [24, 25] where no concrete procedure was specified and as an alternative to the projection techniques presented in [17–21], we propose a simple strategy to avoid the control singularity. Taking into account that a necessary and sufficient condition for the invertibility of the square matrix *S*(*t*) is that det⁡(*S*(*t*)) ≠ 0 or equivalently |det⁡(*S*(*t*))| > 0, we define a positive threshold *μ* in such a way that when |det⁡(*S*(*t*))| ≥ *μ*, the weights of the neural network are updated according to stable learning laws. However, at the instant when the condition |det⁡(*S*(*t*))| < *μ* is presented, the process of learning is immediately stopped. The effect of this modification on the stability of the identification error is thoroughly studied by means of Lyapunov analysis. The proposed strategy is applied to the identification and control of a class of uncertain nonlinear systems with multiple inputs each one subjected to an unknown deadzone.

The deadzone is a nonsmooth nonlinearity commonly found in many practical systems such as electrohydraulic systems [26], pneumatic servo systems [27], DC servo motors [28], and rudders and propellers [29]. When the deadzone is not considered explicitly during the design process, the performance of the control system could be degraded due to an increase of the steady-state error, the presence of limit cycles, or inclusive instability [30–32].

A direct way of compensating the deleterious effect of the deadzone is by calculating its inverse. However, this is not an easy question because in many practical situations, both the parameters and the output of the deadzone are unknown. To overcome this problem, in a pioneer work [30], Tao and Kokotovic proposed to employ an adaptive inverse of the deadzone. This scheme was applied to linear systems in transfer function form. Cho and Bai [33] extended this work and achieved a perfect asymptotic adaptive cancellation of the deadzone. However, their work assumed that the deadzone output was measurable. In [34], the work of Tao and Kokotovic was extended to linear systems in a state space form with nonmeasurable deadzone output. In [35], a new smooth parameterization of the deadzone was proposed and a class of SISO systems with completely known nonlinear functions and with linearly parameterized unknown constants was controlled by using backstepping technique. In order to avoid the construction of the adaptive inverse, in [36], the same class of nonlinear systems as in [35] was controlled by means of a robust adaptive approach and by modeling the deadzone as a combination of a linear term and a disturbance-like term. The controller design in [36] was based on the assumption that maximum and minimum values for the deadzone parameters are a priori known. However, a specific procedure to find such bounds was not provided. Based on the universal approximation property of the neural networks, a wider class of SISO systems in Brunovsky canonical form with completely unknown nonlinear functions and unknown constant control gain was considered in [37–39]. Apparently, the generalization of these results to the case when the control gain is varying and state dependent is trivial. Nevertheless, the solution to this problem is not simple due to the singularity possibility for the control law. In [40, 41], this problem was overcome.

All the aforementioned works about deadzone studied a very particular class of systems, that is, systems in strict Brunovsky canonical form with a unique input. In this paper, we consider a wider class of systems, that is, uncertain nonlinear systems with multiple inputs where each input is preceded by an unknown symmetric deadzone. This global system could be seen as formed by an unknown affine system (see (1)) whose inputs are the outputs of the different deadzones. By generalizing the model used in [36], the multiple deadzones can be represented by means of a diagonal matrix multiplied by the global input vector plus a disturbance-like vector (the diagonal matrix is composed by the unknown symmetric slopes of each deadzone). By using this model, a continuous time recurrent neural network is employed to identify the global unknown dynamics. On the basis of this neural network, an instantaneous mathematical model of the uncertain system can be obtained, and a singularity-free feedback linearization control law is developed in such a way that the system state is compelled to follow a bounded reference trajectory. Once again, by using Lyapunov analysis, the exponential convergence of the tracking error to a bounded zone can be shown. Likewise, the boundedness of all closed-loop signals can be guaranteed.

## 2. Preliminaries 

### 2.1. Notation

Throughout this paper, we will use the following notation.

*I*_*n*×*n*_ represents the identity matrix of dimension *n*. Given *h*(*t*) ∈ *R* ^*n*^, |*h*(*t*)| denotes the Euclidean norm of *h*(*t*); that is, $\left|h\left(t\right)\right|:=\sqrt{h^{T}\left(t\right)h\left(t\right)}$. Given the following vector norm: ||*h*(*t*)||_*∞*_ : = sup⁡_*t*≥0_⁡|*h*(*t*)|, we will say that *h*(*t*) ∈ *L*_*∞*_ when ||*h*(*t*)||_*∞*_ is finite.

### 2.2. Description of the System

In this study, the system to be controlled consists of an unknown multi-input nonlinear plant in which each input is preceded by an unknown symmetric deadzone; that is,

(1) $$\begin{array}{c} \text{PLANT:}\;\;\dot{x}\left(t\right)=f\left(x\left(t\right)\right)+g\left(x\left(t\right)\right)u\left(t\right)+\xi \left(t\right) \end{array}$$

(2) DEADZONE:   ui(t)=DZi(vi(t))={mi(vi(t)−bi,r)vi(t)≥bi,r0bi,l<vi(t)<bi,rmi(vi(t)−bi,l)vi(t)≤bi,l,

where *x*(*t*) ∈ *R* ^*n*^ is the measurable state vector for *t* ∈ *R* ^+^∶ = {*t* : *t* ≥ 0}, *f* : *R* ^*n*^ → *R* ^*n*^ is an unknown but continuous nonlinear vector function, *g* : *R* ^*n*^ → *R* ^*n*×*n*^ is an unknown but continuous nonlinear matrix function, *ξ*(*t*) ∈ *R* ^*n*^ represents an unknown but bounded deterministic disturbance, the *i*th element of the vector *u*(*t*) ∈ *R* ^*n*^, that is, *u*_*i*_(*t*), represents the output of the *i*th deadzone, *v*_*i*_(*t*) is the input to the *i*th deadzone, *b*_*i*,*r*_ and *b*_*i*,*l*_ represent the right and left constant breakpoints of the *i*th deadzone, and *m*_*i*_ is the constant slope of the *i*th deadzone. In accordance with [30, 31], the deadzone model (2) is a static simplification of diverse physical phenomena with negligible fast dynamics.

Note that *v*(*t*) ∈ *R* ^*n*^ is the actual control input to the global system described by (1) and (2). Hereafter, it is considered that the following assumptions are valid.

**Assumption 1 .**

The plant described by (1)-(2) is controllable.

**Assumption 2 .**

The *i*th deadzone output, that is, *u*_*i*_(*t*), is not available for measurement.

**Assumption 3 .**

Although the *i*th deadzone parameters *b*_*i*,*r*_, *b*_*i*,*l*_, and *m*_*i*_ are unknown constants, we can assure that *b*_*i*,*r*_ > 0, *b*_*i*,*l*_ < 0, and *m*_*i*_ > 0 for ∀*i* ∈ {1,2,…*n*}.

### 2.3. Deadzone Representation as a Linear Term and a Disturbance-Like Term

The model of the *i*th deadzone (2) can alternatively be described as follows [34, 42]:

(3) $$\begin{array}{c} u_{i}\left(t\right)=m_{i}v_{i}\left(t\right)+d_{i}\left(t\right), \end{array}$$

where *d*_*i*_(*t*) is given by

(4) $$\begin{array}{c} d_{i}\left(t\right)=\{\begin{array}{cc} -m_{i}b_{i,r} & v_{i}\left(t\right)\geq b_{i,r}\\ -m_{i}v_{i}\left(t\right) & b_{i,l}<v_{i}\left(t\right)<b_{i,r}\\ -m_{i}b_{i,l} & v_{i}\left(t\right)\leq b_{i,l}. \end{array} \end{array}$$

Note that (4) is the negative of a saturation function. Thus, although *d*_*i*_(*t*) could not be exactly known, its boundedness can be assured. Consider that the positive constant ${\overset{-}{d}}_{i}$ is an upper bound for *d*_*i*_(*t*); that is, ${||d_{i}(t)||}_{\infty }\leq {\overset{-}{d}}_{i}$.

Based on (3), in [43], the relationship between *u*(*t*) and *v*(*t*) can be expressed as

(5) $$\begin{array}{c} u\left(t\right)=Mv\left(t\right)+d\left(t\right), \end{array}$$

where *M* : = diag⁡(*m*_1_, *m*_2_,…*m*_*n*_) and *d*(*t*) ∈ *R* ^*n*^ is given by *d*(*t*): = [*d*_1_(*t*),*d*_2_(*t*),…,*d*_*n*_(*t*)]^*T*^. Clearly, *d*(*t*) ∈ *L*_*∞*_. Consider that the positive constant $\overset{-}{d}$ is an upper bound for *d*(*t*).

## 3. Identification Process with Guaranteed Invertibility of *S*(*t*)*ϕ*(*x*(*t*))

In this section, the identification problem of the unknown global dynamics described by (1) and (2) using a recurrent neural network is considered.

Note that an alternative representation for (1) is given by

(6) x˙(t)=Ax(t)+W1∗σ(x(t))+W2∗ϕ(x(t))u(t)+ω(x(t),u(t))+ξ(t),

where *A* ∈ *R* ^*n*×*n*^ is a Hurwitz matrix which can be selected for simplicity as *A* = −*aI*_*n*×*n*_, *a* is a positive constant proposed by the designer, *W*_1_* ∈ *R* ^*n*×*m*^ and *W*_2_* ∈ *R* ^*n*×*n*^ are unknown constant weight matrices, *σ*(·) is an activation vector function with sigmoidal components, that is, *σ*(·)∶ = [*σ*_1_(·),…,*σ*_*m*_(·)]^*⊤*^

(7) σj(x(t))  :=aσj1+exp⁡(−∑i=1ncσj,ixi(t))−dσjfor  j=1,…,m,

where *a*_*σj*_, *c*_*σj*,*i*_, and *d*_*σj*_ are positive constants which can be specified by the designer, *ϕ*(·) : *R* ^*n*^ → *R* ^*n*×*n*^ is a sigmoidal function selected as *ϕ*(·) : = diag⁡(*ϕ*_11_(·), *ϕ*_22_(·),…, *ϕ*_*nn*_(·))

(8) ϕii(x(t))∶=aϕii1+exp⁡(−∑l=1ncϕii,lxl(t))+dϕiifor  i=1,…,n,

where *a*_*ϕii*_, *c*_*ϕii*,*l*_, and *d*_*ϕii*_ are positive constants which can be specified by the designer, and *ω* : *R* ^*n*^ × *R* ^*n*^ → *R* ^*n*^ is the unmodeled dynamics which can be defined simply as

(9) ω(x(t),u(t))∶=f(x(t))+g(x(t))u(t)−Ax(t)−W1∗σ(x(t))−W2∗ϕ(x(t))u(t).

**Remark 4 .**

Note that the structure for the sigmoidal function *ϕ*(*x*(*t*)) was selected in such a way that its invertibility can always be guaranteed.

**Remark 5 .**

Typically, *W*_1_* and *W*_2_* are considered as the optimal values of the weights which minimize the unmodeled dynamics *ω*(*x*(*t*), *u*(*t*)). Although procedures to find such values are presented in [44, 45], in this study the aforementioned values are not required anymore. Thus, the design process is considerably simplified.

**Assumption 6 .**

On a compact set Ω ⊂ *R* ^*n*^, unmodeled dynamics *ω*(*x*(*t*), *u*(*t*)) is bounded by $\overset{-}{\omega }$; that is, ${||\omega \left(x(t),u(t)\right)||}_{\infty }\leq \overset{-}{\omega }$. The disturbance *ξ*(*t*) is also bounded; that is, ||*ξ*(*t*)||_*∞*_ ≤ *Υ*. Both $\overset{-}{\omega }$ and *Υ* are positive constants not necessarily a priori known.

**Assumption 7 .**

The input signal *v*(*t*) is bounded; that is, ${||v(t)||}_{\infty }\leq \overset{-}{v}$, where $\overset{-}{v}$ is a positive constant (not necessarily known).

By substituting (5) into (6), we get

(10) x˙(t)=Ax(t)+W1∗σ(x(t))+W2∗ϕ(x(t))Mv(t)+W2∗ϕ(x(t))d(t)+ω(x(t),u(t))+ξ(t).

**Remark 8 .**

It can be observed that by using the model (5), the actual control input *v*(*t*) appears now directly into the dynamics.

Since, by construction, *ϕ*(*x*(*t*)) is bounded, the term *W*_2_*ϕ(*x*(*t*))*d*(*t*) must also be bounded. Let us define the following expression: *ζ*(*t*): = *W*_2_*ϕ(*x*(*t*))*d*(*t*) + *ω*(*x*(*t*), *u*(*t*)) + *ξ*(*t*). Clearly, this expression is bounded. Let us denote an upper bound for *ζ*(*t*) as $\overset{-}{\zeta }$. This bound is a positive constant not necessarily a priori known. Now, note that the term *W*_2_*ϕ(*x*(*t*))*Mv*(*t*) can be alternatively expressed as S*ϕ(*x*(*t*))*v*(*t*) where S* ∈ *R* ^*n*×*n*^ is an unknown weight matrix. In view of the above, (10) can be rewritten as

(11) $$\begin{array}{c} \dot{x}\left(t\right)=Ax\left(t\right)+W_{1}^{*}\sigma \left(x\left(t\right)\right)+S^{*}\phi \left(x\left(t\right)\right)v\left(t\right)+\zeta \left(t\right). \end{array}$$

Now, consider the following series-parallel structure for a continuous-time recurrent neural network

(12) $$\begin{array}{c} \dot{\hat{x}}\left(t\right)=A\hat{x}\left(t\right)+W_{1}\left(t\right)\sigma \left(x\left(t\right)\right)+S\left(t\right)\phi \left(x\left(t\right)\right)v\left(t\right), \end{array}$$

where ${\hat{x}}_{t}\in R^{n}$ is the state of the neural network, *v*(*t*) ∈ *R* ^*n*^ is the control input as in (10), and *W*_1_(*t*) ∈ *R* ^*n*×*m*^ and *S*(*t*) ∈ *R* ^*n*×*n*^ are the time-varying weight matrices. In order to solve the problem of identifying system (1)-(2) based on the recurrent neural network (12), given the measurable state *x*(*t*) and the input *v*(*t*), we should be able to adjust on line the weights *W*_1_(*t*) and *S*(*t*) by proper learning laws such that the identification error $\Delta (t):=\hat{x}(t)-x(t)$ can be reduced to a bounded zone around zero and, at the same time, the invertibility of *S*(*t*)*ϕ*(*x*(*t*)) can be guaranteed. Specifically, we employ in this study the following learning laws:

(13) $$\begin{array}{c} {\overset{\cdot }{W}}_{1}\left(t\right)=-\gamma \left(t\right)\left(k_{1}\Delta \left(t\right)\sigma^{T}\left(x\left(t\right)\right)+l_{1}W_{1}\left(t\right)\right), \end{array}$$

(14) $$\begin{array}{c} \overset{\cdot }{S}\left(t\right)=-\gamma \left(t\right)\left(k_{2}\Delta \left(t\right)v^{T}\left(t\right)\phi^{T}\left(x\left(t\right)\right)+l_{2}S\left(t\right)\right), \end{array}$$

where *k*_1_, *l*_1_, *k*_2_, and *l*_2_ are positive constants which can be selected by the designer,

(15) $$\begin{array}{c} \gamma \left(t\right)=\{\begin{array}{cc} 1, & \text{if}\;\;\left|\det\left(S\left(t\right)\right)\right|\geq \mu ,\\ 0, & \text{otherwise}, \end{array} \end{array}$$

and *μ* is a positive constant adjustable by the designer.

Based on the learning laws (13) and (14), we can establish here the following result.

**Theorem 9 .**

If Assumptions 2, 3, 6, and 7 are satisfied, the constant *a* is selected greater than 1.5, and the weight matrices *W*_1_(*t*), *S*(*t*) of the neural network (12) are adjusted by the learning laws (13) and (14), respectively, then

(a) the identification error and the weights of the neural network (12) are bounded as follows:
  (16) $$\begin{array}{c} \Delta \left(t\right),W_{1}\left(t\right),S\left(t\right)\in L_{\infty }, \end{array}$$
(b1) when *γ*(*t*) = 1, the norm of the identification error, that is, $\left|\hat{x}(t)-x(t)\right|$, converges exponentially fast to a zone bounded by the term
  (17) $$\begin{array}{c} \sqrt{\frac{2\beta_{1}}{\alpha_{1}}}, \end{array}$$
 where *α*_1_ : = min⁡{(2*a* − 1), *l*_1_, *l*_2_} and
  (18) $$\begin{array}{c} \beta_{1}∶=\frac{1}{2}{\overset{-}{\zeta }}^{2}+\frac{l_{1}}{2k_{1}}\mathrm{tr}\left\{W_{1}^{*T}W_{1}^{*}\right\}+\frac{l_{2}}{2k_{2}}\mathrm{tr}\left\{S^{*T}S^{*}\right\}, \end{array}$$
(b2) when *γ*(*t*) = 0, the identification error is uniformly ultimately bounded with respect to the set
  (19) $$\begin{array}{c} Y=\left\{\hat{x}\left(t\right)-x\left(t\right):=\left|\hat{x}\left(t\right)-x\left(t\right)\right|\leq \sqrt{\frac{\beta_{2}}{\alpha_{2}}}\right\}, \end{array}$$
 where *α*_2_ : = *a* − (3/2) and *β*_2_ is an upper bound for the term
  (20) 12|(W1(t)−W1∗)σ(x(t))|2  +12|(S(t)−S∗)ϕ(x(t))v(t)|2+12ζ−.

**Proof of Theorem 9.**

First, let us determine the dynamics of the identification error. The first derivative of Δ(*t*) is simply

(21) $$\begin{array}{c} \dot{\Delta }\left(t\right)=\dot{\hat{x}}\left(t\right)-\dot{x}\left(t\right). \end{array}$$

Substituting (12) and (11) into (21) yields the following:

(22) Δ˙t=Ax^(t)+W1(t)σ(x(t))+S(t)ϕ(x(t))v(t)−Ax(t)−W1∗σ(x(t))−S∗ϕ(x(t))v(t)−ζ(t)=AΔ(t)+W~1(t)σ(x(t))+S~(t)ϕ(x(t))v(t)−ζ(t),

where ${\tilde{W}}_{1}(t):=W_{1}(t)-W_{1}^{*}$ and $\tilde{S}(t):=S(t)-S^{*}$.

Consider the following Lyapunov function candidate:

(23) V(t)=12ΔT(t)Δ(t)+12k1tr⁡{W~1T(t)W~1(t)}+12k2tr⁡{S~T(t)S~(t)}.

The first derivative of *V*(*t*) is

(24) V·(t)=ΔT(t)Δ˙(t)+1k1tr⁡{W~˙1T(t)W~1(t)}+1k2tr⁡{S~˙T(t)S~(t)}.

Substituting (22) into (24) and taking into account that *A* = −*aI*_*n*×*n*_ yield

(25) V·(t)=−a|Δ(t)|2+ΔT(t)W~1(t)σ(x(t))+ΔT(t)S~(t)ϕ(x(t))v(t)−ΔT(t)ζ(t)+1k1tr⁡{W~˙1T(t)W~1(t)}+1k2tr⁡{S~˙T(t)S~(t)}.

Since ${\tilde{W}}_{1}(t):=W_{1}(t)-W_{1}^{*}$ and $\tilde{S}(t):=S(t)-S^{*}$, the first derivatives for ${\dot{\tilde{W}}}_{1}(t)$ and $\dot{\tilde{S}}(t)$ are clearly ${\dot{\tilde{W}}}_{1}(t)={\dot{W}}_{1}(t)$ and $\dot{\tilde{S}}(t)=\dot{S}(t)$, respectively. However, ${\dot{W}}_{1}(t)$ and $\dot{S}(t)$ are given by the learning laws (13) and (14). Therefore, by substituting (13) into ${\dot{\tilde{W}}}_{1}(t)={\dot{W}}_{1}(t)$ and (14) into $\dot{\tilde{S}}(t)=\dot{S}(t)$ and the corresponding expressions into the right-hand side of (25), it is possible to obtain

(26) V·(t)=−a|Δ(t)|2+ΔT(t)W~1(t)σ(x(t))+ΔT(t)S~(t)ϕ(x(t))v(t)−ΔT(t)ζ(t)+γ(t)tr⁡{−σ(x(t))ΔT(t)W~1(t)}−γ(t)l1k1tr⁡{W1T(t)W~1(t)}+γ(t)tr⁡{−ϕ(x(t))v(t)ΔT(t)S~(t)}−γ(t)l2k2tr⁡{ST(t)S~(t)}.

It is easy to show that

(27) $$\begin{array}{c} -\Delta^{T}\left(t\right)\zeta \left(t\right)\leq \frac{1}{2}{\left|\Delta \left(t\right)\right|}^{2}+\frac{1}{2}{\left|\zeta \left(t\right)\right|}^{2}\leq \frac{1}{2}{\left|\Delta \left(t\right)\right|}^{2}+\frac{1}{2}{\overset{-}{\zeta }}^{2}. \end{array}$$

Substituting (27) into (26) yields

(28) V·(t)≤−a|Δ(t)|2+ΔT(t)W~1(t)σ(x(t))+ΔT(t)S~(t)ϕ(x(t))v(t)+12|Δ(t)|2+12ζ−2+γ(t)tr⁡{−σ(x(t))ΔT(t)W~1(t)}−γ(t)l1k1tr⁡{W1T(t)W~1(t)}+γ(t)tr⁡{−ϕ(x(t))v(t)ΔT(t)S~(t)}−γ(t)l2k2tr⁡{ST(t)S~(t)}.

Let us consider the two possible cases for *γ*(*t*) separately.

*Case I (when γ*(*t*) = 1). On this condition, taking into account that

(29) tr⁡{−σ(x(t))ΔT(t)W~1(t)}=−tr⁡{σ(x(t))ΔT(t)W~1(t)}=−tr⁡{ΔT(t)W~1(t)σ(x(t))}=−ΔT(t)W~1(t)σ(x(t))tr⁡{−ϕ(x(t))v(t)ΔT(t)S~(t)}  =−tr⁡{ϕ(x(t))v(t)ΔT(t)S~(t)}  =−tr⁡{ΔT(t)S~(t)ϕ(x(t))v(t)}  =−ΔT(t)S~(t)ϕ(x(t))v(t)

if (29) is substituted into (28) and reducing the like terms, we can get

(30) V·(t)≤−a|Δ(t)|2+12|Δ(t)|2+12ζ−2−l1k1tr⁡{W1T(t)W~1(t)}−l2k2tr⁡{ST(t)S~(t)}.

Besides, it can be proven [46] that

(31) tr⁡{W1T(t)W~1(t)}=12tr⁡{W1T(t)W1(t)}+12tr⁡{W~1T(t)W~1(t)}−12tr⁡{W1∗TW1∗},tr⁡{ST(t)S~(t)}=12tr⁡{ST(t)S(t)}+12tr⁡{S~T(t)S~(t)}−12tr⁡{S∗TS∗}.

Substituting (31) into (30) yields

(32) V·(t)≤−a|Δ(t)|2+12|Δ(t)|2+12ζ−2−l12k1tr⁡{W1T(t)W1(t)}−l12k1tr⁡{W~1T(t)W~1(t)}+l12k1tr⁡{W1∗TW1∗}−l22k2tr⁡{ST(t)S(t)}−l22k2tr⁡{S~T(t)S~(t)}+l22k2tr⁡{S∗TS∗}

or

(33) V·(t)≤−(2a−1){12|Δ(t)|2}−l1(12k1tr⁡{W~1T(t)W~1(t)})−l2(12k2tr⁡{S~T(t)S~(t)})+12ζ−2+l12k1tr⁡{W1∗TW1∗}+l22k2tr⁡{S∗TS∗}.

In view of

(34) $$\begin{array}{c} \alpha_{1}∶=\min\left\{\left(2a-1\right),l_{1},l_{2}\right\},\\ \beta_{1}∶=\frac{1}{2}{\overset{-}{\zeta }}^{2}+\frac{l_{1}}{2k_{1}}\mathrm{tr}\left\{W_{1}^{*T}W_{1}^{*}\right\}+\frac{l_{2}}{2k_{2}}\mathrm{tr}\left\{S^{*T}S^{*}\right\}, \end{array}$$

the following bound as a function of *V*(*t*) can finally be determined for $\overset{\cdot }{V}(t)$:

(35) $$\begin{array}{c} \overset{\cdot }{V}\left(t\right)\leq -\alpha_{1}V\left(t\right)+\beta_{1}. \end{array}$$

This implies that the following bound for *V*(*t*) can be established (the demonstration of this intermediate result can be consulted in [43])

(36) $$\begin{array}{c} V\left(t\right)\leq V\left(0\right)\exp\left(-\alpha_{1}t\right)+\frac{\beta_{1}}{\alpha_{1}}\left(1-\exp\left(-\alpha_{1}t\right)\right). \end{array}$$

Since by definition *α*_1_ and *β*_1_ are positive constants, the right-hand side of the inequality (36) can be bounded by *V*(0) + (*β*_1_/*α*_1_). Thus, *V*(*t*) ∈ *L*_*∞*_ and since by construction *V*(*t*) is a nonnegative function, the boundedness of Δ(*t*), ${\tilde{W}}_{1}(t)$, and $\tilde{S}(t)$ can be guaranteed. Because *W*_1_* and S* are bounded, $W_{1}(t)={\tilde{W}}_{1}(t)+W_{1}^{*}$ and $S(t)=\tilde{S}(t)+S^{*}$ must be bounded too and the first part of Theorem 9 has been proven. With respect to the second part of this theorem, from (23), it is evident that (1/2)|Δ(*t*)|^2^ ≤ *V*(*t*). Taking into account this fact and from (36), we get

(37) $$\begin{array}{c} \left|\Delta \left(t\right)\right|\leq \sqrt{2V\left(0\right)\exp\left(-\alpha_{1}t\right)+\frac{2\beta_{1}}{\alpha_{1}}\left(1-\exp\left(-\alpha_{1}t\right)\right)}. \end{array}$$

By taking the limit as *t* → *∞* of the inequality (37), we can guarantee that |Δ(*t*)| converges exponentially fast to a zone bounded by the term $\sqrt{2\beta_{1}/\alpha_{1}}$ and the part (b1) of the Theorem 9 has been proven.

*Case II (when γ*(*t*) = 0). Under this condition, (28) becomes simply

(38) V·(t)≤−a|Δ(t)|2+ΔT(t)W~1(t)σ(x(t))+ΔT(t)S~(t)ϕ(x(t))v(t)+12|Δ(t)|2+12ζ−2.

It is easy to show that

(39) $$\begin{array}{c} \Delta^{T}\left(t\right){\tilde{W}}_{1}\left(t\right)\sigma \left(x\left(t\right)\right)\leq \frac{1}{2}{\left|\Delta \left(t\right)\right|}^{2}+\frac{1}{2}{\left|{\tilde{W}}_{1}\left(t\right)\sigma \left(x\left(t\right)\right)\right|}^{2},\\ \Delta^{T}\left(t\right)\tilde{S}\left(t\right)\phi \left(x\left(t\right)\right)v\left(t\right)\leq \frac{1}{2}{\left|\Delta \left(t\right)\right|}^{2}+\frac{1}{2}{\left|\tilde{S}\left(t\right)\phi \left(x\left(t\right)\right)v\left(t\right)\right|}^{2}. \end{array}$$

Substituting (39) into (38) yields

(40) V·(t)≤−(a−32)|Δ(t)|2+12|W~1(t)σ(x(t))|2+12|S~(t)ϕ(x(t))v(t)|2+12ζ−2.

Now, from the learning laws (13) and (14), the condition *γ*(*t*) = 0 implies that ${\overset{\cdot }{W}}_{1}(t)=0$ and $\overset{\cdot }{S}(t)=0$. This means that *W*_1_(*t*) and *S*(*t*) become constant matrices and consequently they are bounded. From the above and taking into account Assumption 7, we can conclude the boundedness of the term

(41) $$\begin{array}{c} \frac{1}{2}{\left|{\tilde{W}}_{1}(t)\sigma \left(x(t)\right)\right|}^{2}+\frac{1}{2}{\left|\tilde{S}(t)\phi \left(x(t)\right)v(t)\right|}^{2}+\frac{1}{2}\overset{-}{\zeta }. \end{array}$$

Consider that a bound for such term is the constant (not necessarily known) *β*_2_ and given *α*_2_ : = *a* − (3/2), (40) becomes

(42) $$\begin{array}{c} \overset{\cdot }{V}\left(t\right)\leq -\alpha_{2}{\left|\Delta \left(t\right)\right|}^{2}+\beta_{2}. \end{array}$$

Note that if $\left|\Delta (t)\right|>\sqrt{\beta_{2}/\alpha_{2}}$, then $\dot{V}(t)<0$. This means that *V*(*t*) < *V*(0) for ∀*t* ≥ 0 and consequently |Δ(*t*)| ∈ *L*_*∞*_. If $\left|\Delta (t)\right|\leq \sqrt{\beta_{2}/\alpha_{2}}$ clearly |Δ(*t*)| is bounded. Thus, the uniform ultimate boundedness of |Δ(*t*)| with respect to the set

(43) $$\begin{array}{c} Y=\left\{\hat{x}\left(t\right)-x\left(t\right):=\left|\hat{x}\left(t\right)-x\left(t\right)\right|\leq \sqrt{\frac{\beta_{2}}{\alpha_{2}}}\right\} \end{array}$$

can be guaranteed and the proof is completed.

**Remark 10 .**

Note that the utilization of *γ*(*t*) permits to guarantee that det⁡(*S*(*t*)) ≠ 0 for ∀*t* ≥ 0. Hence, *S*(*t*) is an invertible matrix for ∀*t* ≥ 0. Certainly, the designer should select *S*(0) in such a way that this condition can be fulfilled.

## 4. Tracking Controller 

In this section, an appropriate control law *v*(*t*) will be determined in such a way that the state *x*(*t*) of system (1)-(2) follows a given reference trajectory *x*_*r*_(*t*), and, at the same time, all closed-loop signals stay bounded.

**Assumption 11 .**

The reference trajectory *x*_*r*_(*t*) and its first derivative ${\dot{x}}_{r}(t)$ are continuous and bounded. Besides, these variables are available for the design.

By simultaneously adding and subtracting the terms *W*_1_(*t*)*σ*(*x*(*t*)) and *S*(*t*)*ϕ*(*x*(*t*))*v*(*t*) into (11), we can get

(44) x˙(t)=Ax(t)+W1(t)σ(x(t))+S(t)ϕ(x(t))v(t)+W1∗σ(x(t))−W1(t)σ(x(t))+S∗ϕ(x(t))v(t)−S(t)ϕ(x(t))v(t)+ζ(t)=Ax(t)+W1(t)σ(x(t))+S(t)ϕ(x(t))v(t)−W~1(t)σ(x(t))−S~(t)ϕ(x(t))v(t)+ζ(t).

Let us define the term *δ*(*t*) as

(45) $$\begin{array}{c} \delta \left(t\right):=-{\tilde{W}}_{1}\left(t\right)\sigma \left(x\left(t\right)\right)-\tilde{S}\left(t\right)\phi \left(x\left(t\right)\right)v\left(t\right)+\zeta \left(t\right). \end{array}$$

According to this definition, (44) can be expressed simply as

(46) $$\begin{array}{c} \dot{x}\left(t\right)=Ax\left(t\right)+W_{1}\left(t\right)\sigma \left(x\left(t\right)\right)+S\left(t\right)\phi \left(x\left(t\right)\right)v\left(t\right)+\delta \left(t\right). \end{array}$$

Note that if the learning laws (13) and (14) are used to adjust the weights *W*_1_(*t*) and *S*(*t*), respectively, then according to Theorem 9 ${\tilde{W}}_{1}(t)$ and $\tilde{S}(t)$ belong to *L*_*∞*_. As ${\tilde{W}}_{1}(t)\sigma \left(x(t)\right)$ and $\tilde{S}(t)\phi \left(x(t)\right)v(t)$ are known and bounded terms and *ζ*(*t*) is an unknown but bounded term, from (45), we can conclude that *δ*(*t*) is an unknown but bounded term. Consider that the positive constant (not necessarily known) $\overset{-}{\delta }$ is an upper bound for *δ*(*t*); that is, ${||\delta (t)||}_{\infty }\leq \overset{-}{\delta }$.

Now, let us define the tracking error *ε*(*t*) as

(47) $$\begin{array}{c} \varepsilon \left(t\right):=x\left(t\right)-x_{r}\left(t\right). \end{array}$$

The first derivative of (47) is simply

(48) $$\begin{array}{c} \dot{\varepsilon }\left(t\right)=\dot{x}\left(t\right)-{\dot{x}}_{r}\left(t\right). \end{array}$$

Substituting (46) into (48) yields

(49) ε˙(t)=Ax(t)+W1(t)σ(x(t))+S(t)ϕ(x(t))v(t)+δ(t)−x˙r(t).

By using the principle of feedback linearization, we propose the following control law:

(50) v(t)=(S(t)ϕ(x(t)))−1×{−Ax(t)−W1(t)σ(x(t))+x˙r(t)+Cε(t)},

where *C* is a Hurwitz matrix which can be selected by simplicity as *C* = −*cI*_*n*×*n*_, *c* is a positive constant proposed by the designer such that *c* > 0.5. If (50) is substituted into (49), we can get

(51) $$\begin{array}{c} \dot{\varepsilon }\left(t\right)=C\varepsilon \left(t\right)+\delta \left(t\right). \end{array}$$

We can analyze the dynamics of the tracking error *ε*(*t*) given in (51) by proposing the following Lyapunov function candidate:

(52) $$\begin{array}{c} V_{2}\left(t\right)=\frac{1}{2}\varepsilon^{T}\left(t\right)\varepsilon \left(t\right). \end{array}$$

The first derivative of (52) is

(53) $$\begin{array}{c} {\dot{V}}_{2}\left(t\right)=\varepsilon^{T}\left(t\right)\dot{\varepsilon }\left(t\right). \end{array}$$

Substituting (51) into (53) yields

(54) $$\begin{array}{c} {\dot{V}}_{2}\left(t\right)=\varepsilon^{T}\left(t\right)C\varepsilon \left(t\right)+\varepsilon^{T}\left(t\right)\delta \left(t\right). \end{array}$$

It is easy to show that

(55) $$\begin{array}{c} \varepsilon^{T}\left(t\right)\delta \left(t\right)\leq \frac{1}{2}{\left|\varepsilon \left(t\right)\right|}^{2}+\frac{1}{2}{\left|\delta \left(t\right)\right|}^{2}\leq \frac{1}{2}{\left|\varepsilon \left(t\right)\right|}^{2}+\frac{1}{2}{\overset{-}{\delta }}^{2}. \end{array}$$

Taking into account (55) and given *C* = −*cI*_*n*×*n*_, (54) becomes

(56) $$\begin{array}{c} {\dot{V}}_{2}\left(t\right)\leq -c{\left|\varepsilon (t)\right|}^{2}+\frac{1}{2}{\left|\varepsilon (t)\right|}^{2}+\frac{1}{2}{\overset{-}{\delta }}^{2} \end{array}$$

or

(57) $$\begin{array}{c} {\dot{V}}_{2}\left(t\right)\leq -\left(2c-1\right)\frac{1}{2}{\left|\varepsilon \left(t\right)\right|}^{2}+\frac{1}{2}{\overset{-}{\delta }}^{2}. \end{array}$$

By defining the following positive constants *α*_3_ : = 2*c* − 1 and $\beta_{3}:=\left(1/2\right){\overset{-}{\delta }}^{2}$, (57) can be expressed as

(58) $$\begin{array}{c} {\dot{V}}_{2}\left(t\right)\leq -\alpha_{3}V_{2}\left(t\right)+\beta_{3}. \end{array}$$

Hence,

(59) $$\begin{array}{c} V_{2}\left(t\right)\leq V_{2}\left(0\right)\exp\left(-\alpha_{3}t\right)+\frac{\beta_{3}}{\alpha_{3}}\left(1-\exp\left(-\alpha_{3}t\right)\right). \end{array}$$

According to (52) and (59), it can be established that

(60) $$\begin{array}{c} \left|\varepsilon \left(t\right)\right|\leq \sqrt{{\left|\varepsilon \left(0\right)\right|}^{2}\exp\left(-\alpha_{3}t\right)+\frac{2\beta_{3}}{\alpha_{3}}\left(1-\exp\left(-\alpha_{3}t\right)\right)}. \end{array}$$

From this last inequality, the boundedness of |*ε*(*t*)| can be concluded. From the above and taking into account that *ε*(*t*) = *x*(*t*) − *x*_*r*_(*t*) and as, according to Assumption 11, *x*_*r*_(*t*) is bounded, *x*(*t*) must also be bounded. This implies, according to (50), that *v*(*t*) belongs to *L*_*∞*_ and this last result agrees with Assumption 7. Finally, by taking the limit as *t* → *∞* in both sides of the inequality (60), we can guarantee that |*ε*(*t*)| converges exponentially fast to a zone bounded by the term $\sqrt{2\beta_{3}/\alpha_{3}}$. In this way, the following theorem has been proven.

**Theorem 12 .**

If Assumptions 1–11 are satisfied, the constant *c* is selected greater than 0.5, the weight matrices *W*_1_(*t*), *S*(*t*) of the neural network (12) are adjusted by the learning laws (13) and (14), and the control law (50) is applied to the system formed by (1)-(2), then

(a) the tracking error and the state of system (1) are bounded
  (61) $$\begin{array}{c} \varepsilon \left(t\right),x\left(t\right)\in L_{\infty }, \end{array}$$
(b) the norm of the tracking error, that is, |*x*(*t*) − *x*_*r*_(*t*)|, converges exponentially fast to a zone bounded by the term
  (62) $$\begin{array}{c} \sqrt{\frac{2\beta_{3}}{\alpha_{3}}}, \end{array}$$
 where *α*_3_ : = 2*c* − 1 and $\beta_{3}:=\left(1/2\right){\overset{-}{\delta }}^{2}$.

## 5. Numerical Example

In order to illustrate the strategy proposed in this paper, a simulation example is presented in this section. A second order nonlinear system (see (63)) is used as the unknown plant. Consider

(63) x˙1(t)=−2x1(t)+x2(t)+x1(t)x2(t)+x2(t)exp⁡(−2x1(t))+(sin⁡(x1(t)+x2(t))+2)u1(t),x˙2(t)=−3x2(t)+x1(t)cos⁡(x2(t))+(x12(t)+x22(t)+0.5)u2(t).

**Figure 1:**
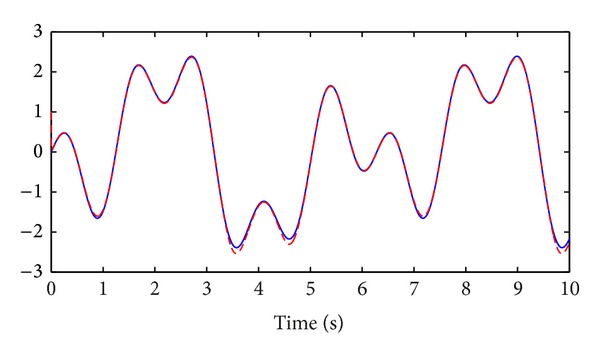
Tracking process: reference trajectory *x*_*r*,1_(*t*), solid line; system state *x*_1_(*t*), dashed line.

**Figure 2:**
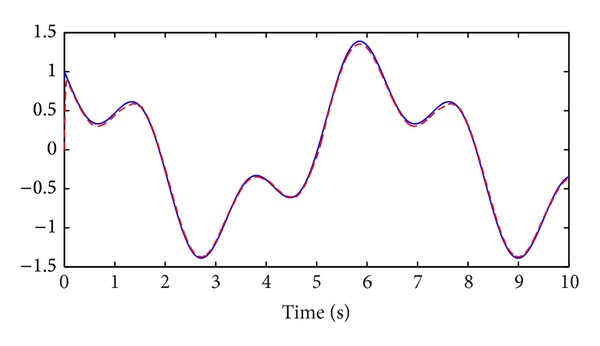
Tracking process: reference trajectory *x*_*r*,2_(*t*), solid line; system state *x*_2_(*t*), dashed line.

**Figure 3:**
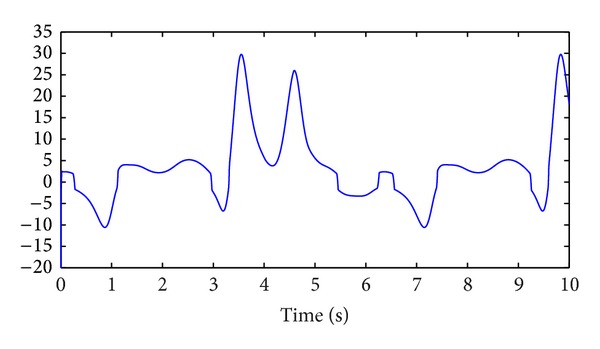
Control signal *v*_1_(*t*).

**Figure 4:**
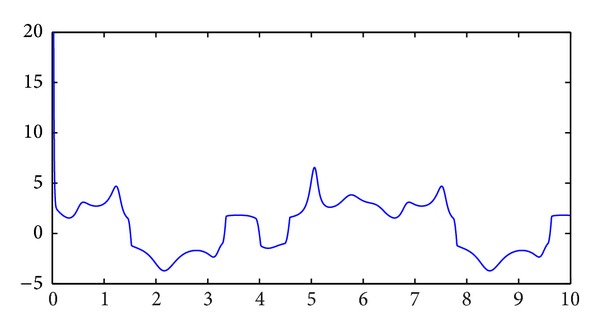
Control signal *v*_2_(*t*).

The initial condition for system (63) is *x*_1_(0) = 1, *x*_2_(0) = 0. Each input of this system is preceded by a deadzone. The parameters of each deadzone are as follows: *m*_1_ = 1.3, *b*_1,*r*_ = 2, *b*_1,*l*_ = −1.5, *m*_2_ = 0.8, *b*_2,*r*_ = 1.5, and *b*_2,*l*_ = −1. The states of the reference trajectory *x*_*r*_(*t*) are selected as *x*_*r*,1_(*t*) = sin(*t*) − 1.5sin(2*t*) + sin(5*t*) and *x*_*r*,2_(*t*) = cos⁡(*t*) − 0.5sin(3*t*). The parameters of the neural identifier and the control law are selected by trial and error as *a* = 5, ${\hat{x}}_{1}(0)=1$, ${\hat{x}}_{2}(0)=0$, *k*_1_ = 100, *l*_1_ = 10, *k*_2_ = 120, *l*_2_ = 5, *σ*_1_(*x*(*t*)) = 2/(1 + exp⁡(−*x*_1_(*t*))) − 1, *σ*_2_(*x*(*t*)) = 2/(1 + exp⁡(−*x*_2_(*t*))) − 1, *ϕ*_11_(*x*(*t*)) = 1/(1 + exp⁡(−*x*_1_(*t*))) + 0.3, *ϕ*_22_(*x*(*t*)) = 1/(1 + exp⁡(−*x*_2_(*t*))) + 0.3, and *c* = 1500. The results of the tracking process are presented in Figures 1 and 2 for the first 10 seconds. In Figure 1, the state *x*_1_(*t*) of the nonlinear system (63) is represented by dashed line whereas the reference trajectory *x*_*r*,1_(*t*) is represented by solid line. The state *x*_2_(*t*) (dashed line) and the reference trajectory *x*_*r*,2_(*t*) (solid line) are presented in Figure 2 for comparison. The control signals *v*_1_(*t*) and *v*_2_(*t*) are shown in Figures 3 and 4, respectively.

## 6. Conclusions

In this paper, the exponential tracking for a class of nonlinear systems with unknown deadzones using recurrent neural networks was considered. Since physical model is not available, the neural networks are used to identify the unknown dynamics. The main novelty in this study is a systematic procedure for the modification of the learning laws of the synaptic weights in such a way that the avoidance of the control singularity can be guaranteed. This objective is achieved by continuously monitoring the determinant of the coupling matrix or more specifically the input weight matrix. By defining a threshold for the determinant of the input weight, a “dangerous” region next to the singularity can be established. When such region is reached, the learning process is immediately stopped. In this way, the invertibility of the coupling matrix is guaranteed. The effect of this modification on the identification error stability is rigorously studied by means of Lyapunov analysis. On the basis of the instantaneous mathematical model obtained by the identification process, a singularity-free feedback linearization control law is developed in order to compel the system state to follow a reference trajectory. By means of Lyapunov-like analysis, the exponential convergence of the tracking error to a bounded zone can be proven. Likewise, the boundedness of all closed-loop signals can be guaranteed. Certainly, the main attractiveness of the suggested approach is its simplicity. However, it must be mentioned that the turning off of the learning law could reduce the system performance. In fact, in such conditions, the control action becomes mainly proportional.
